# Screening for autoimmune diseases in apparently healthy antinuclear antibody positive individuals

**DOI:** 10.3389/fmed.2024.1455673

**Published:** 2024-08-20

**Authors:** Rama Andraos, Awais Ahmad, Lina Wirestam, Charlotte Dahle, Martina Frodlund, Johan Rönnelid, Alf Kastbom, Christopher Sjöwall

**Affiliations:** ^1^Division of Inflammation and Infection/Rheumatology, Department of Biomedical and Clinical Sciences, Linköping University, Linköping, Sweden; ^2^Division of Inflammation and Infection/Clinical Immunology & Transfusion Medicine, Department of Biomedical and Clinical Sciences, Linköping University, Linköping, Sweden; ^3^Department of Immunology, Genetics and Pathology, Uppsala University, Uppsala, Sweden

**Keywords:** autoantibodies, autoimmune disease, interferon-α, healthy blood donors, systemic lupus erythematosus, Sjögren’s disease

## Abstract

**Background:**

Anti-nuclear antibodies (ANA) assessed by immunofluorescence (IF) microscopy are associated with systemic autoimmune rheumatic diseases (SARD) and can be detected years before onset of clinical symptoms. Recent data indicate dysregulation of the immune system with increased levels of proinflammatory cytokines, including type I interferons (IFN), in ANA-positive versus ANA-negative individuals. Herein, the aims were to investigate IF-ANA, ANA fine specificities, and IFN-α protein levels in relation to self-reported symptoms, as well as clinical signs, of SARD in a large group of healthy blood donors (HBD).

**Methods:**

Sera from 825 HBD (48.8% females) were included. IF-ANA was assessed, using HEp-2 cells, according to the routine at the accredited laboratory of Clinical Immunology, Linköping University Hospital. All samples were analyzed for IgG-ANA fine specificities using addressable laser bead assay (ALBIA) at the same laboratory. IFN-α was determined using ELISA. Antibody-positive individuals, and their sex- and age-matched antibody-negative controls, were asked to fill a questionnaire regarding symptoms associated with SARD.

**Results:**

In total, 130 HBD (15.8%) were positive with IF-ANA and/or ALBIA. Anti-U1RNP was significantly more common among women. Generally, self-reported symptoms correlated poorly with IF-ANA and/or ALBIA results. Two females with high levels of Ro60/SSA, Ro52/SSA and IFN-α reported mild sicca symptoms and were diagnosed with Sjögren’s disease after clinical evaluation.

**Conclusion:**

A considerable proportion of apparently HBD are autoantibody positive, but without clear association to self-reported symptoms. Nevertheless, the combination of autoantibodies, relevant symptoms and high IFN-α levels identified the small proportion of individuals with SARD in the study population.

## Introduction

The anti-nuclear antibody (ANA)-associated systemic autoimmune rheumatic diseases (SARD), which among others include systemic lupus erythematosus (SLE), Sjögren’s disease (SjD), systemic sclerosis (SSc), idiopathic inflammatory myopathies, mixed and undifferentiated connective tissue diseases, are chronic multisystem autoimmune diseases with a significant morbidity and mortality (1). A hallmark of their pathogenesis constitutes loss of tolerance which leads to autoreactivity and production of antibodies against self-nuclear antigens (2). Similarly to many autoantibodies, ANA can be detected in serum many years before onset of clinical symptoms, representing a phase of subclinical autoimmunity and the levels may fluctuate over time in established SARD (3–6). Still, the cut-off pursued for a positive ANA test is essential.

An “abnormal titer” of ANA assessed by immunofluorescence (IF) microscopy (IF-ANA) is one of the 11 classification criteria for SLE according to the 1982 American College of Rheumatology (ACR-82) whereas the 2012 Systemic Lupus International Collaborating Clinics (SLICC-12) criteria state that an ANA test “above the laboratory reference value” remains a criterion for SLE (7, 8). The importance of ANA was further highlighted in the most recent SLE criteria set from 2019 where the European Alliance of Associations for Rheumatology/ACR classification introduce ANA as an entry criterion, but with the recommendation of using HEp-2 cells or a solid-phase ANA screening immunoassay (9). Only the most recent classification ground for SLE states how to define the cut-off level for ANA (9).

Considerable efforts have been made to better understand the mechanisms that drive autoimmune disease. Compared to subjects with SLE, ANA-positive healthy individuals show lower levels of stem cell factor, B lymphocyte stimulator, and type I interferons (IFN) as well as higher levels of IL-1 receptor antagonist (10, 11). Nevertheless, some data indicate that the immune system is already dysregulated in ANA-positive versus ANA-negative healthy individuals, i.e., elevation of proinflammatory cytokines in serum and altered proportions of monocytes, B cells and T_H_ follicular cells (12).

Of etiopathogenetic relevance, activation of the type I IFN response constitutes a common denominator of several SARD which is also demonstrated by shared positivity for several ANA fine specificities (13, 14). Recent data from randomized controlled trials have shown that blocking the type I IFN receptor by anifrolumab decreases global disease activity in SLE and led to the approval of anifrolumab in both US and Europe (15). Still, the role of IFNs in disease initiation is not entirely clear (16). IFN activity is usually quantified using expression of interferon-stimulated genes (ISG) or by IFN-α ELISA. El-Sherbiny et al. described two continuous ISG expression scores that provided clinically meaningful differences in IFN status between and within autoimmune diseases (17). By adding family history of SARD to ISG expression data, prediction of SARD onset was improved compared to ISG expression alone in an at-risk cohort (18).

The aims of the current study were to investigate IF-ANA, ANA fine specificities, and IFN-α protein levels in relation to self-reported symptoms, as well as clinical signs, of SARD in a large group of blood donors.

## Materials and methods

### Study population

We included serum samples from 825 consecutive and apparently healthy blood donors (HBD), comprising 403 females (48.8%) with a median age of 46 years (range 18–71) and 422 (51.2%) males with a median age of 42 years (range 19–77) from one blood donation center in Linköping, Sweden, during the period March 2018 to June 2019. Only three individuals (0.4%) were ≥ 70 years of age (19). The samples were stored at −70°C until analyses were performed.

### Indirect IF microscopy

IF-ANA was analyzed according to the routine at the accredited laboratory of Clinical Immunology, Linköping University Hospital, Sweden, using Olympus microscope BX43, lens 20X/0.75 Plan Super Apochromat, illumination with LED diode (CoolLed pE-100, wavelength 470 nm) set at 50% of maximal light intensity, multi-spot slides with fixed HEp-2 cells (ImmunoConcepts, Sacramento, CA, United States) as antigen substrate, and fluorescein isothiocyanate (FITC)-conjugated γ-chain-specific anti-human IgG dilution 1:200 as detection antibody (Dako A/S, Glostrup, Denmark). The cut-off level for a positive IF-ANA test was set at titer 800, corresponding to the 95th percentile (“abnormal titer”) among 420 HBD (260 women, median age 54 years, range 19–89; 160 men, median age 46 years, range 19–72) according to international recommendations for ANA analysis (5.7% IF-ANA positive, 19 women and 5 men) (20). The serum samples which had previously been used in verification of IF-ANA analysis did not originate from the same individuals as the HBD of the current study population. Results included interpretation of the staining patterns using the International Consensus on ANA Patterns (ICAP) nomenclature (20).

### ANA fine specificities

All samples were analyzed for IgG-ANA fine specificities, including antibodies against double-stranded DNA (anti-dsDNA) and thirteen other autoantibody specificities, by FIDIS™ Connective Profile interpreted with the Solinium software version 1.7.1.0 (both from Theradiag, Croissy-Beaubourg, France) at the Clinical Immunology Laboratory, Linköping University Hospital as previously detailed (21). This addressable laser bead assay (ALBIA) simultaneously measures autoantibodies to Ro52/SSA, Ro60/SSA, La/SSB, Smith antigen (Sm), Smith/ribonucleoprotein complex (Sm/RNP), U1-RNP, dsDNA, Scl70, Jo1, centromere B (CENP-B), ribosomal P protein (RiboP), histone, PmScl and proliferating cell nuclear antigen (PCNA). The manufacturer’s recommended cut-off >40 units per ml (U/ml) was used for all fine specificities.

### IFN-α assay

IFN-α was analyzed by ELISA according to the manufacturer’s instructions [Human IFN-α (pan-specific) ELISA^PRO^ kit], Mabtech, Nacka Strand, Sweden (14). This ELISA detects subtypes 1/13, 2, 4, 5, 6, 7, 8, 10, 14, 16 and 17 of IFN-α with a standard ranging from 5 to 4,000 pg./mL.

### Questionnaire

Antibody positive individuals (positive with either IF and/or with detected fine specificities; *n* = 130) as well as their individually sex- and age-matched antibody negative control from the same cohort (*n* = 130) were asked to fill in an unvalidated symptom questionnaire in Swedish with 14 questions (Supplementary Table S1). The questionnaire was constructed to identify both overt and subtle symptoms potentially associated with SARD, and it was delivered to the donors with regular post service up to 3 months after blood sampling.

### Clinical assessment

All blood donors testing ANA-positive on IF, and/or showing any positive ANA fine specificity, in combination with relevant self-reported symptoms of rheumatic disease in the questionnaire were offered a visit to an experienced rheumatologist at Linköping University Hospital, Region Östergötland (C.S.). A full clinical assessment was performed and, if clinically indicated, additional blood tests (e.g., antiphospholipid antibodies, complement proteins, direct Coombs’ test, anti-C1q antibodies as well as autoimmune liver disease- and myositis-associated antibodies), radiology, sialometry and/or biopsies were ordered to rule out any suspicion of SARD.

### Statistics

The data were analyzed using SPSS statistics software V.27.0 (IBM) and Prism V.9 (GraphPad Software, La Jolla, United States) for construction of graphs. Differences between groups were calculated using χ^2^ or Fisher’s exact test where appropriate, and with the Mann–Whitney *U* test. *p* values of <0.05 were considered statistically significant.

### Ethics considerations

Oral and written informed consent was obtained from all included subjects. The study was conducted according to the Declaration of Helsinki, and the study protocol was approved by the Regional Ethics Board in Linköping (Decision no. 2017/474-31).

## Results

In total, 130 of the 825 blood donors (15.8%) showed at least one positive test using IF microscopy (7.2%) or ALBIA (9.9%). Only 11 (8.5%) out of the 130 autoantibody positive HBD showed a combined positivity for IF-ANA and ALBIA. The mean age of the positive individuals was 43.8 years (range 19–68) and 62/130 (47.7%) were women.

Table 1 shows the most common ANA staining patterns with AC-1 (homogenous) and AC-1, −4, −5 (homogenous/speckled) dominating. The mean age of the positive individuals was 42.8 years (range 19–67) and 36/59 (61%) were women. No significant associations between age and staining pattern were observed but individuals with AC-8, −9, −10 (nucleolar) tended to be younger, and those with AC-4, −5 (speckled) were slightly older, than other blood donors. Overall, IF-ANA positivity was more common among female blood donors but it did not reach statistical significance (*p* = 0.052).

**Table 1:** ANA staining patterns with ICAP nomenclature in relation to mean age and sex among the 825 healthy blood donors.

ICAP	Pattern	HEp-2 positive (*n*)	Positive (%)	Mean age (years)	Female sex (%)
AC-1	Homogenous	20	2.4	41.5	80
AC-1, -4, -5	Homogenous/Speckled	18	2.2	43.0	50
AC-4, -5	Speckled	11	1.3	47.3	55
AC-6	Nuclear dots	1	0.1	59.0	100
AC-8, -9, -10	Nucleolar	9	1.1	37.8	44
Positive	Any	59	7.2	42.8	61

As demonstrated in Table 2, 82 of 825 blood donors showed positivity on at least one ANA fine specificity using ALBIA. The mean age of the positive individuals was 45.1 years (range 21–68) and 34/82 (41.5%) were women. Ro60/SSA, U1-RNP, PmScl and PCNA were the most frequently observed specificities. Whereas Ro60/SSA, PmScl and PCNA were numerically most common among men, only U1-RNP positivity reached a statistically significant difference (*p* = 0.014) being more common in women. Subjects with antibodies against dsDNA and PCNA tended to be younger than those positive for other ANA fine specificities but not reaching statistical significance.

**Table 2:** ANA fine specificities (ALBIA) in relation to mean age and sex among the 825 healthy blood donors.

ANA fine specificity	Positive, *n* (%)	Mean age (years)	Positive, *n* (%)	Females, *n* (%)	Males, *n* (%)	*p* value
dsDNA	7 (0.8)	36.9	7 (0.8)	5 (71.4)	2 (28.6)	0.23
Ro60/SSA	16 (1.9)	48.9	16 (1.9)	5 (31.3)	11 (68.8)	0.15
Ro52/SSA	5 (0.6)	48.4	5 (0.6)	3 (60)	2 (40)	0.25
La/SSB	4 (0.5)	53.0	4 (0.5)	1 (25)	3 (75)	0.34
Sm	0	–	0	0	0	∞
SmRNP	1 (0.1)	25.0	1 (0.1)	0	1 (100)	∞
U1-RNP	15 (1.8)	43.6	15 (1.8)	12 (80)	3 (20)	**0.014**
Scl70	3 (0.4)	52.0	3 (0.4)	0	3 (100)	∞
Jo1	5 (0.6)	43.6	5 (0.6)	2 (40)	3 (60)	0.69
CENP-B	2 (0.2)	49.5	2 (0.2)	0	2 (100)	∞
RiboP	2 (0.2)	28.5	2 (0.2)	1 (50)	1 (50)	0.97
Histone	2 (0.2)	46.0	2 (0.2)	1 (50)	1 (50)	0.97
PmScl	18 (2.2)	50.6	18 (2.2)	6 (33.3)	12 (66.7)	0.18
PCNA	13 (1.6)	35.7	13 (1.6)	4 (30.8)	9 (69.2)	0.19
≥ 1 specificity	82 (9.9)	45.1	82 (9.9)	34 (41.5)	48 (58.5)	0.16
> 1 specificity	5 (0.6)	45.8	5 (0.6)	2 (40)	3 (60)	0.69
Negative	743 (90.1)	42.3	743 (90.1)	369 (91.6)	374 (88.6)	0.16

Bold value indicates that statistical significance was reached; i.e., U1-RNP positivity was more common among female HBD.

**Figure 1:**
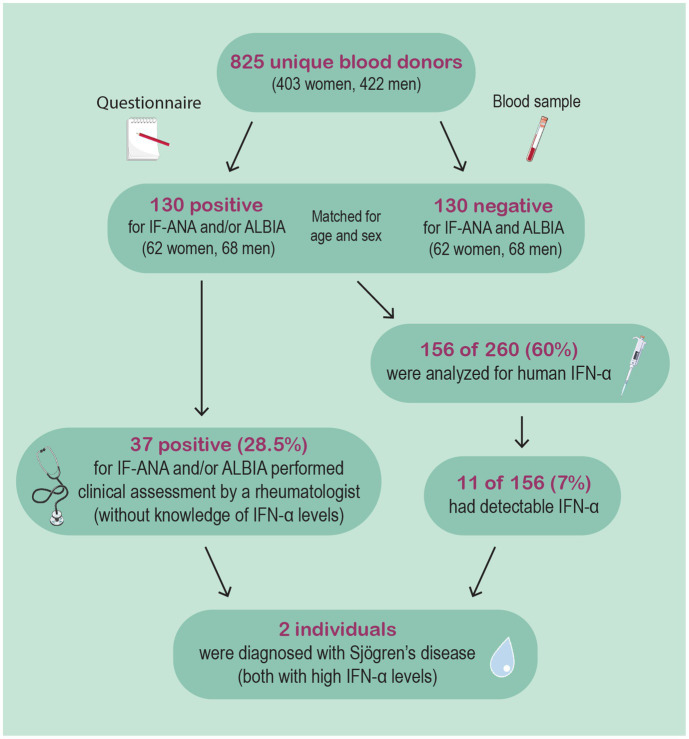
This flow-chart illustrates the study procedure. 825 unique blood donors were included, whereof 130 were deemed IF-ANA and/or ALBIA positive. 37 subjects were clinically evaluated by an experienced rheumatologist based on autoantibody findings and relevant self-reported symptoms. Eventually, two females were diagnosed with Sjögren’s disease. Both were later found to have high IFN-α levels.

As illustrated in the flow chart (Figure 1), IFN-α protein levels were assessed among 156/260 (60%) matched samples (i.e., 52 antibody positive and 104 antibody negative samples). The mean level of IFN-α protein in the autoantibody positive subgroup was 25.3 versus 17.6 pg./mL (*p* = 0.04) in the autoantibody negative group. This comparison refers only to samples with quantifiable levels (i.e., ≥5 pg./mL), which were 3/52 in the antibody positive and 8/104 in the antibody negative group (not significant). All three subjects in the autoantibody positive group with quantifiable IFN-α protein levels were females, whereas 50% were men in the autoantibody negative group.

**Figure 2:**
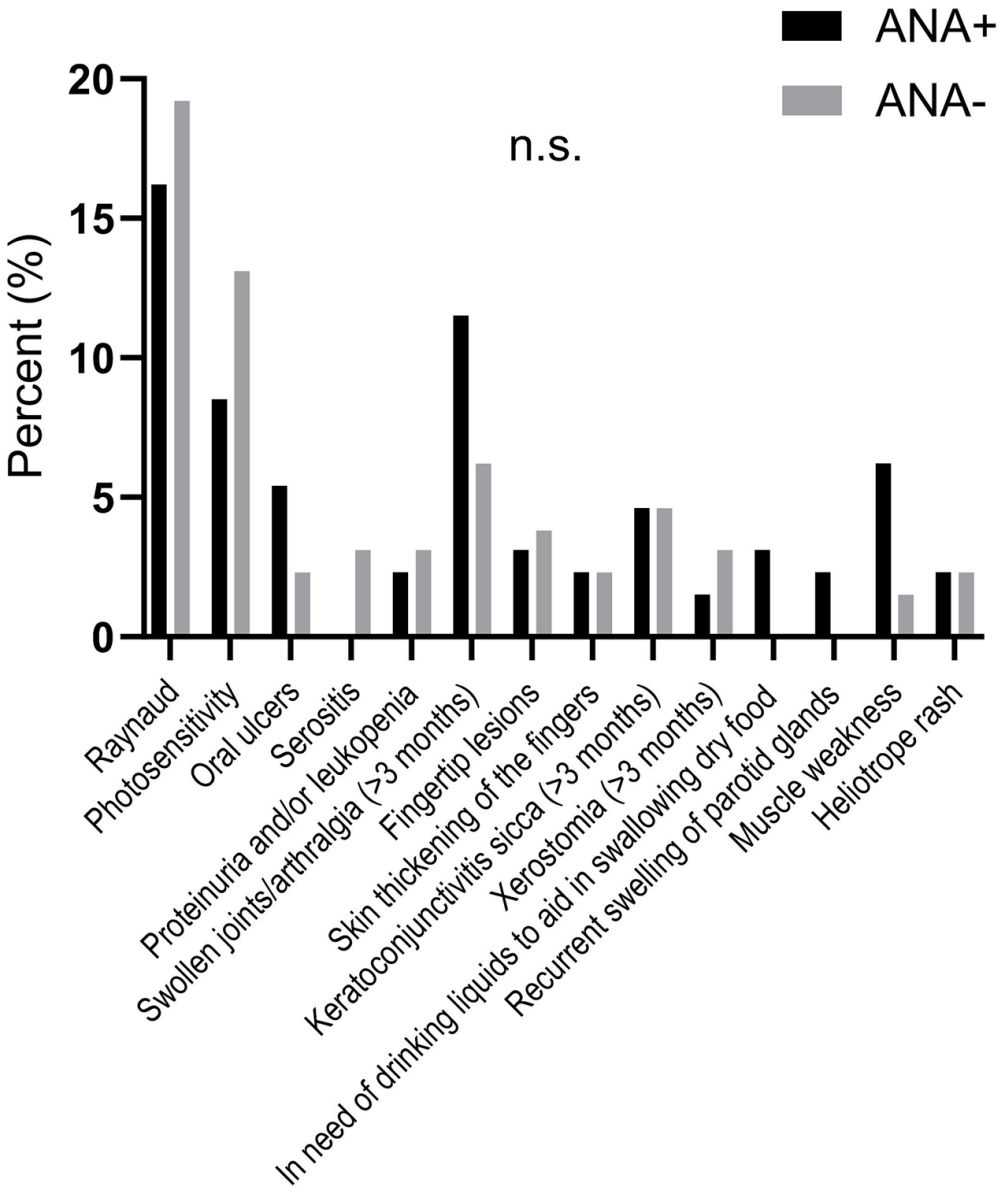
The graph shows self-reported symptoms among IF-ANA and/or ALBIA positive versus negative blood donors. No significant differences were observed.

Among the antibody positive blood donors, 125 (96.2%) agreed to answer the questionnaire (Supplementary Table S1). Among those not responding, 4 of 5 (80%) were men. Subsequently, 125 individually sex- and age-matched autoantibody negative controls from the same cohort also answered the questionnaire. As demonstrated in Figure 2, “swollen joints and/or arthralgia” as well as “muscle weakness” were numerically more common among antibody positive blood donors whereas “Raynaud” and “photosensitivity” as well as serositis and xerostomia were more frequently reported by antibody negative controls but without reaching statistical significances.

Based on the combination of self-reported symptoms and autoantibody findings, 37 subjects (28.5%) were offered (and accepted) a clinical assessment at the Rheumatology unit, Linköping University Hospital. This offer was not based on results of the IFN-α measurement as it had not yet been performed at the time. In total, based on the clinical evaluation, results of IF microscopy and ALBIA tests, and sialometry, two individuals were diagnosed with SjD. These two females, representing 1.5% of the IF-ANA and/or ALBIA positive blood donor population, eventually showed the highest assessed levels of IFN-α protein levels of all investigated subjects. Both were IF-ANA positive (AC-4, −5; speckled pattern) and showed strong positivity for Ro60/SSA as well as for Ro52/SSA.

## Discussion

Individuals who are accepted as blood donors in Sweden are highly selected based on their subjective health. In the current study, we aimed to assess autoantibody specificities using established techniques and stringent cut-offs in a large cohort of apparently healthy donors. Our main findings were that >15% of HBD test positive with IF-ANA (7.2%) and/or ALBIA (9.9%), using the manufacturer’s recommended cut-offs. Only 11 of 825 HBD showed a combined positivity, probably illustrating the different conformational forms of antigens in cell- versus bead-based assays.

In line with our findings, Kim et al. recently reported that the most common fine specificities among ANA positive blood donors were SSA and U1-RNP (18, 22). We further observed that IF-ANA positivity, as well as anti-dsDNA and anti-U1-RNP antibody positivity, were more common among female blood donors. Anti-U1-RNP is often associated with Raynaud’s phenomenon in females with SARD, but Raynaud is also frequently reported in the general population (14, 23). However, on a group level in the current study, we found that self-reported symptoms potentially associated with SARD appeared to correlate rather poorly with the autoantibody findings, similarly to prior reports using smaller study populations, underlining the low diagnostic specificity of a positive IF-ANA test (24).

IFN-α levels are raised in many patients with SLE and elevated levels of type I IFNs constitute a characteristic feature of several SARD and may predict progression to disease in ANA positive individuals (13, 14, 18, 22). Inspired by these observations, we asked whether the diagnostic accuracy could be increased by adding information on activation of the type I IFN system to autoantibody results. Indeed, we could show that IFN-α protein levels were higher among IF-ANA and/or ALBIA positive blood donors. In total, two female blood donors (0.24%) who reported mild sicca symptoms displayed elevated levels of anti-SSA antibodies (Ro60 and Ro52) and were eventually diagnosed with SjD, and found to have high levels of serum IFN-α.

Previous studies have had similar focus as ours. A recent paper by Brunekreef et al. from Netherlands, using data from electronic health records, concluded that progression to a connective tissue disease (CTD) is uncommon in individuals with a history of a positive IF-ANA test. They found that 16 of 1,030 (1.6%) ANA positive subjects received a CTD diagnosis (SLE being most common) within a mean time from the blood draw to diagnosis of approximately 2.3 years (25). This is in line with our current findings.

Selmi et al. assessed a cohort from the general population in Northern Italy and found that >18% were IF-ANA positive, with decreasing percentages having higher titers. In line with our findings, the female predominance was found to be lower compared to those with overt CTD (26). Importantly, the authors observed no associations with cancer or mortality. However, their finding of >18% ANA positivity appears to be high but is in line with what we reported 16 years ago in blood donors when a cut-off screening dilution of 1:60 was applied (27). Obviously, using such cut-off in clinical routine will introduce specificity issues and could not be considered as an “abnormal titer” of ANA (7, 8). Similarly to the definition of a positive rheumatoid factor test according to the 1987 ACR classification criteria for rheumatoid arthritis (RA), we advocate a cut-off level of >95th percentile among HBD to define an abnormal level of ANA analyzed by indirect IF microscopy utilizing fixed HEp-2 cells as source of nuclear antigens and, importantly, γ-chain specific secondary antibodies to pinpoint immunoglobulin (Ig)G-class IF-ANA (28–30). The use of the 95th percentile specificity and the use of IgG-specific detection antibodies is in agreement with the international recommendations for ANA testing (20).

Nowadays, it is well established that autoantibodies may precede SARD (3, 4, 6). The samples for the current study were obtained between 2018 and 2019. Hitherto, only two of the 825 blood donors have received a SARD diagnosis by a rheumatologist at Region Östergötland during 5 years of follow-up. Although this constitutes a substantial timeframe from a clinical perspective, we cannot exclude that additional donors will develop overt SARD over time. A clear limitation of the current study is the cross-sectional nature with only one sample per blood donor since it is known that the levels of autoantibodies may fluctuate over time, although anti-Ro/SSA and anti-La/SSB levels have been shown to be more stable over time in contrast to anti-dsDNA and anti-Sm (5, 27). Based on available data, we cannot determine if the antibody findings are persistent or not. A follow-up study with new sampling including collection of new self-reported symptoms would be desirable.

In addition, we did not have information on family history of SARD. IFN-α activity has shown a complex heritable trait based on data from healthy family members of patients with SLE (31). Unfortunately, only 156/260 (60%) HBD samples were available for IFN-α analysis. The major strength of the current study is the large study population with self-reported symptoms. In addition, 37/130 (28.5%) autoantibody positive subjects were evaluated clinically at the Rheumatology unit where a diagnosis of SjD eventually was confirmed in two female blood donors. A limitation worth mentioning is that we did not have available data on environmental factors, e.g., smoking habits or use of hormones, particularly contraceptives, with potential association to antibody positivity, organ manifestations and the progression to SARD (32–39).

To conclude, our data revealed that a considerable proportion of apparently healthy blood donors are autoantibody positive. IF-ANA and/or ALBIA positivity associated poorly with self-reported symptoms. However, the combination of autoantibodies, relevant symptoms and high IFN-α levels identified the small proportion of individuals with SARD among blood donors.

## Data Availability

The original contributions presented in the study are included in the article/Supplementary material, further inquiries can be directed to the corresponding authors.
