# Supplementary material for: Screening for autoimmune diseases in apparently healthy antinuclear antibody positive individuals
Source: Front Med (Lausanne). 2024 Aug 20;11:1455673. doi: 10.3389/fmed.2024.1455673 (PMC11368755; doi:10.3389/fmed.2024.1455673)
Supplement: Supplementary file 1 [file Table_1.docx]

**SUPPLEMENTARY TABLE 1**

| 1 | Do your fingers turn white when exposed to cold temperature? |
| --- | --- |
| 2 | Have you developed a rash, with or without skin ulcers, when you have been exposed to sunlight? (NOTE! Not ”regular” sunburn) |
| 3 | Have you developed mouth ulcers that took long time to heal? (NOTE! Not oral herpes) |
| 4 | Have you been diagnosed with pleuritis or pericarditis? |
| 5 | Has it been noted that you have protein in your urine, or a low number of white blood cells in your blood? |
| 6 | Have you had swollen and/or painful joints that lasted for more than 3 months? |
| 7 | Have you developed ulcers or scars on your fingertips that were not related to external injury? |
| 8 | Do you feel that the skin on your fingers is thickened and tight? |
| 9 | Have you had dry eyes for more than 3 months? |
| 10 | Have you had dry mouth for more than 3 months? |
| 11 | Do you often need to drink extra to swallow food? |
| 12 | Have your salivary glands been swollen for a long time (weeks)? |
| 13 | Do you experience weakness in the thigh muscles, such as makes you unable to stand up without using hands or arms for support? |
| 14 | Have you noticed swelling around the eyes, or purple skin rashes on the eyelids and/or around the eyes? |

The questionnaire that was sent out to the blood donors was in Swedish. This table represents a translation.
